# Influence of methane seepage on isotopic signatures in living deep-sea benthic foraminifera, 79° N

**DOI:** 10.1038/s41598-022-05175-1

**Published:** 2022-01-21

**Authors:** Katarzyna Melaniuk, Kamila Sztybor, Tina Treude, Stefan Sommer, Tine L. Rasmussen

**Affiliations:** 1 Centre of Arctic Gas Hydrate, Environment and Climate, Department of Geosciences, UiT The Arctic University of Norway, Tromsø, Norway; 2 Akvaplan-Niva AS, Fram Centre, Tromsø, Norway; 3 Department of Earth, Planetary, and Space Sciences, University of California Los Angeles, Los Angeles, USA; 4 Department of Atmospheric and Oceanic Sciences, University of California Los Angeles, Los Angeles, USA; 5 GEOMAR Helmholtz Centre for Ocean Research Kiel, Kiel, Germany

**Keywords:** Microbiology, Environmental sciences, Ocean sciences

## Abstract

Fossil benthic foraminifera are used to trace past methane release linked to climate change. However, it is still debated whether isotopic signatures of living foraminifera from methane-charged sediments reflect incorporation of methane-derived carbon. A deeper understanding of isotopic signatures of living benthic foraminifera from methane-rich environments will help to improve reconstructions of methane release in the past and better predict the impact of future climate warming on methane seepage. Here, we present isotopic signatures (δ^13^C and δ^18^O) of foraminiferal calcite together with biogeochemical data from Arctic seep environments from c. 1200 m water depth, Vestnesa Ridge, 79° N, Fram Strait. Lowest δ^13^C values were recorded in shells of *Melonis barleeanus*, − 5.2‰ in live specimens and − 6.5‰ in empty shells, from sediments dominated by aerobic (MOx) and anaerobic oxidation of methane (AOM), respectively. Our data indicate that foraminifera actively incorporate methane-derived carbon when living in sediments with moderate seepage activity, while in sediments with high seepage activity the poisonous sulfidic environment leads to death of the foraminifera and an overgrowth of their empty shells by methane-derived authigenic carbonates. We propose that the incorporation of methane-derived carbon in living foraminifera occurs via feeding on methanotrophic bacteria and/or incorporation of ambient dissolved inorganic carbon.

## Introduction

One of the consequences of the ongoing climate warming is an increase in ocean temperature^1^. The Arctic is already warming about twice as fast as the global average, because of a process called ‘the polar amplification’ caused by decline in sea-ice cover and increased atmospheric heat transport from the equator to the Arctic. As large amounts of methane are stored on Arctic continental margins in the form of gas hydrates (pressure–temperature sensitive methane captured in ice^2–4^), concern has increased that ongoing ocean warming will trigger destabilization of the gas hydrate reservoirs and cause further release of methane in the future^1,3,5,6^. Because methane is a ~ 25 times more potent greenhouse gas than CO_2_, a significant increase in the atmosphere can cause further amplification of the global warming. In the geological past, methane released from marine reservoirs has been suggested to be linked to paleoclimatic and palaeoceanographic changes during the Quaternary^7^, Late Paleocene^8^, the Cretaceous^9^ and also been linked to the Permian–Triassic extinction event^10^. In methane-rich environments such as cold seeps, the carbon pool available for benthic foraminifera is enriched in inorganic methane-derived CO_2_ and HCO_3_^−^, and organic carbon in the form of methane-related microbial communities characterized by low δ^13^C values.

It has been hypothesized that benthic foraminifera are able to record past methane seepage events by incorporating the low δ^13^C values derived from methane into their shells (called tests), and that they thus have a high potential to record variations in past methane release from the seabed^11,12^. Although the δ^13^C signatures of benthic foraminifera are a widely used proxy in paleoceanography to reconstruct past ocean circulation and productivity^13–15^, it is still disputed how methane-derived carbon enters foraminiferal shells, which might be via consumption of ^13^C-depleted microbes, the presence of microbial symbionts^16,17^, active incorporation of dissolved inorganic carbon (DIC) from ambient seawater, or as a result of passive diagenetic alteration via deposition of methane-derived authigenic carbonates (MDAC) from anaerobic oxidation of methane^18^.

Improved understanding of isotopic signatures of benthic foraminifera as a consequence of methane-related biological and geochemical processes is necessary to develop more robust models for the interpretation of past as well as for the prediction of future methane release and the impact on climate. Modern cold seeps, i.e., methane-fuelled chemosynthetic ecosystems^19^, are reasonable analogs of past methane-rich environments^20–22^, and thus are perfect environments to study the impact of methane seepage on the isotopic signatures of living benthic foraminifera. For this study, we investigated the stable isotopes (δ^13^C and δ^18^O) of modern and dead (= empty shells without protoplasm; see “Methods”) benthic foraminiferal species from Vestnesa Ridge, ~ 79° N, 7° E, northwestern Svalbard margin in the eastern Fram Strait. Vestnesa Ridge is known for very active seepage of thermogenic methane gas from a deep reservoir^23^.

## Study area

**Figure 1:**
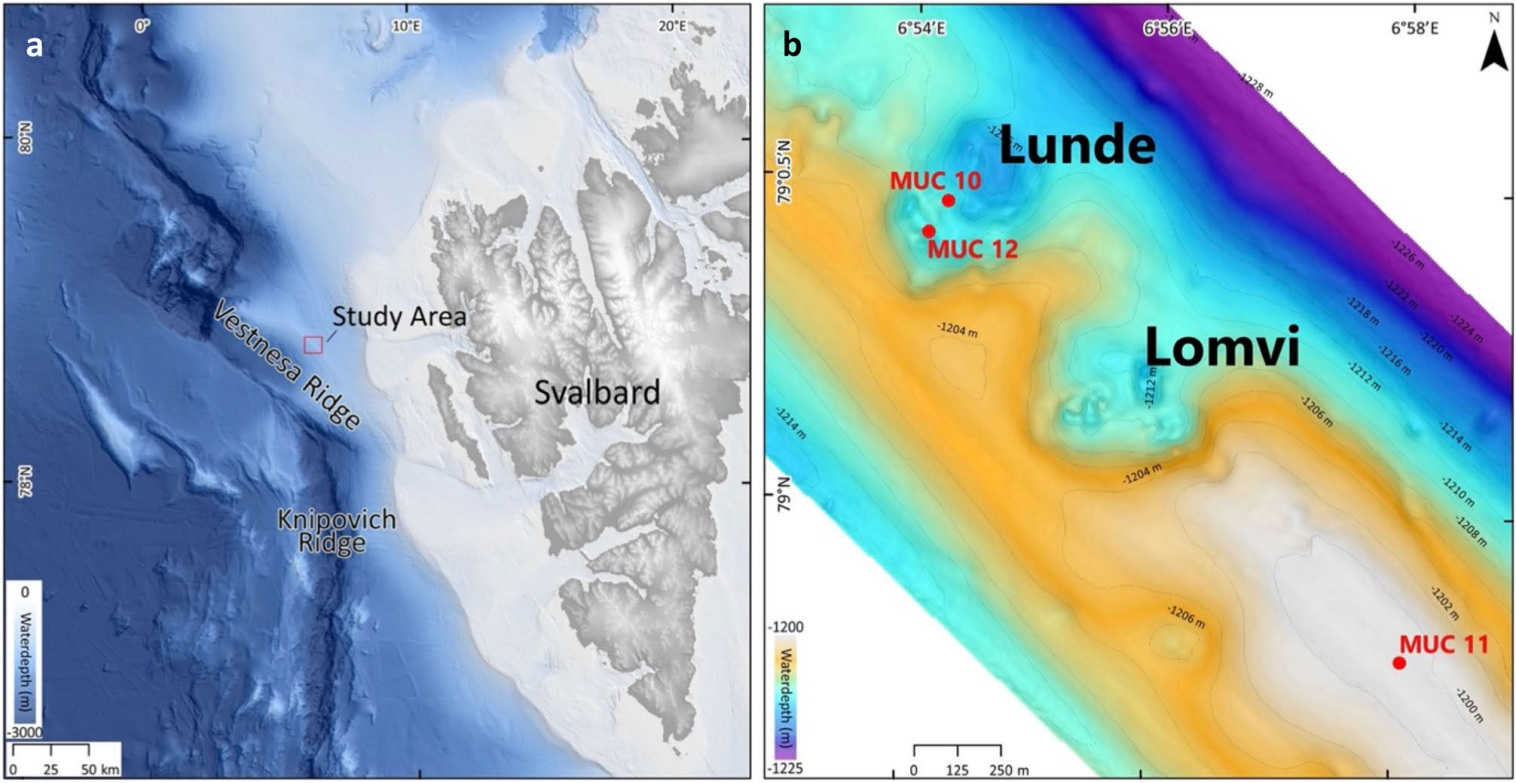
(**a**) Svalbard margin in the Eastern Fram Strait (bathymetry from Jakobsson et al.^33^). (**b**) Detail of Vestnesa Ridge (modified from Bünz et al.^23^). Red dots indicate multicorer locations: *Siboglinidae* field (MUC 10), bacterial mats field (MUC 12), and control site (MUC 11). This figure is original, made using ArcMap v10.6. https://www.esri.com/en-us/arcgis/products/arcgis-desktop/overview".

Vestnesa Ridge is located at water depths of 1200–1300 m at ~ 79° N, 5–7° E in the eastern Fram Strait, NW of Svalbard (Fig. 1a). It is ~ 100 km long and surrounded by ∼1-km thick sediment drifts of Pliocene–Pleistocene age. The crest of the ridge shows a series of pockmarks (i.e., shallow seabed depressions) through which methane-rich fluids actively seep from gas-hydrate and deep, free-gas reservoirs^23,24^. Fluid flow and methane seepage probably started in the early Pleistocene^25^. The two most active pockmarks at Vestnesa Ridge are informally referred to as ‘Lomvi’ and ‘Lunde’^23^ (Fig. 1b). Previous paleo-studies from ‘Lomvi’ pockmark revealed chemosymbiotic fossil macrofaunal communities related to the different types of seep environments^26^ (and references therein), and studies of fossil foraminifera showed diagenetic alterations of their tests^12,18^. Biological investigations from this pockmark documented the presence of species-rich live macro- and megafaunas^27,28^, carbonate outcrops^12,29^, and heterogeneous environmental conditions associated with methane release. Also, living benthic foraminifera from ‘Lomvi’ pockmark were previously investigated^30–32^; nevertheless, the isotopic signatures of investigated specimens indicated no influence of methane. For the present study, the neighboring pockmark ‘Lunde’ was selected. This site is slightly less active compared to ‘Lomvi’ and sediments less disturbed^12,18,23^. Two multicorer (MUC) stations were sampled by video guidance. MUC 10 targeted a dense field of *Siboglinidae* (chemosymbiotic tube worms) and MUC 12 targeted a field of sulfur-bacterial mats (Fig. 1; Table S2). A third MUC station (MUC 11) was selected outside the ‘Lunde’ pockmark to serve as a reference site without methane seepage (see “Methods” for details). Pore water chemistry was determined in MUC 10 and 12.

## Results

### Sediment biogeochemistry

**Figure 2:**
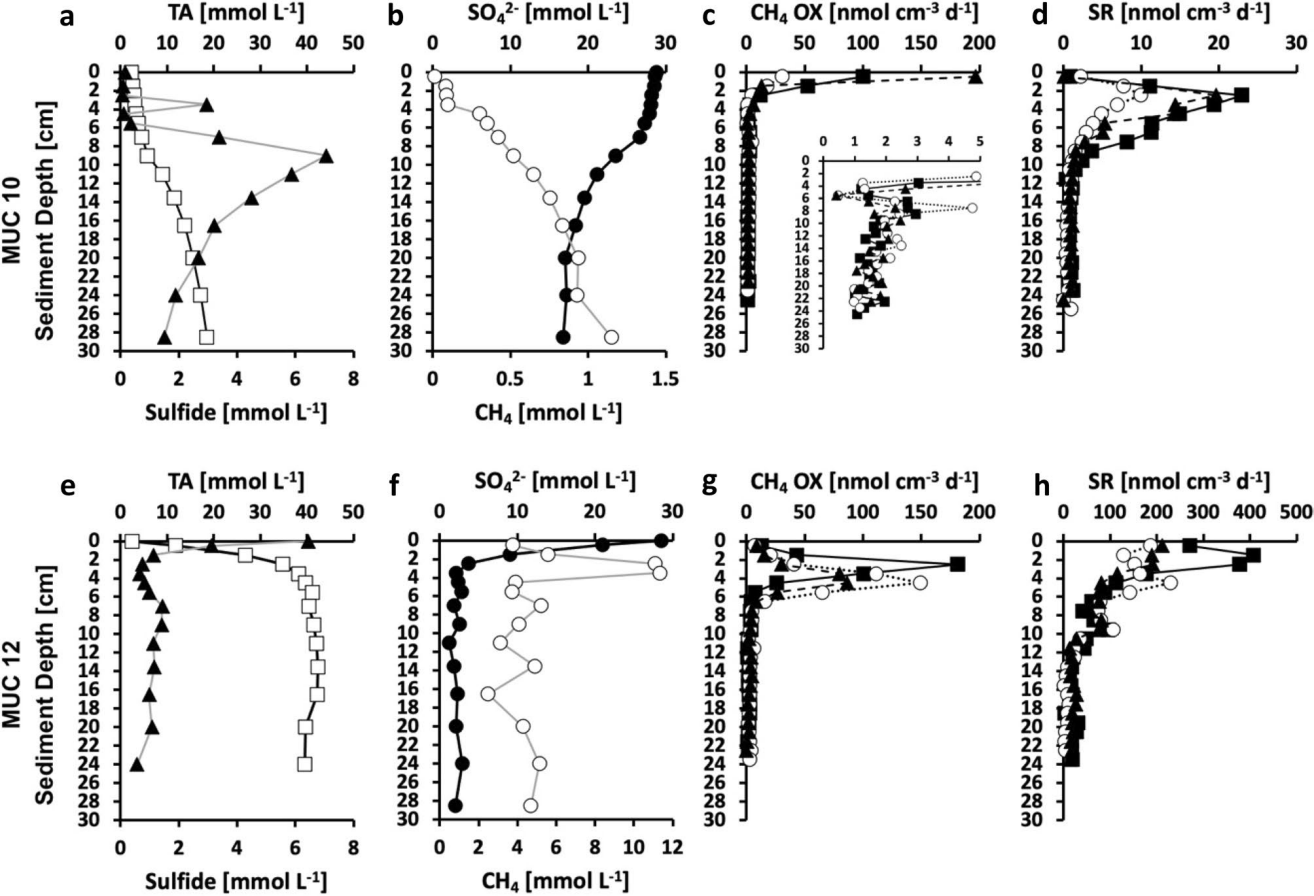
Biogeochemical data of sediment from MUC 10 (*Siboglinidae* field*,* **a**–**d**) and MUC 12 (bacterial mat field, **e**–**h**). (**a**,**e**) Concentrations of pore-water total alkalinity (TA, open squares), and sulfide (solid triangles). (**b**,**f**) Concentrations of pore-water sulfate (SO_4_^2−^, solid circles) and sediment methane (CH_4_, open circles). Note the different x-axes for methane. (**c**,**g**) Methane oxidation rates (CH_4_ OX, symbols represent three replicates). Note that c includes an insert that focusses on rates < 5 nmol cm^−3^ d^−1^. (**d**,**h**) Sulfate reduction rates (SR, symbols represent three replicates).

Sediments of the *Siboglinidae* field MUC 10 showed strong indications for bio-irrigation by the tubeworms (Fig. 2a,b). Sulfate (~ 28 mmol L^−1^), total alkalinity (~ 3 mmol L^−1^), sulfide (< 0.2 mmol L^−1^, except 3 mmol L^−1^ at 3–4 cm), and methane concentrations (< 0.1 mmol L^−1^) remained relatively unchanged in the topmost 4–6 cm. Irrigation was further suggested by the bright brown coloring of the top sediment layers (Fig. S1) indicative of oxidized sediment. Below 4–6 cm, sulfate decreased while total alkalinity, sulfide, and methane increased (Fig. 2a,b). Sulfate declined to a minimum of 16.8 mmol L^−1^ at the bottom of the core, while total alkalinity and methane increased to 18.5 and 1.1 mmol L^−1^, respectively. Sulfide peaked with 7 mmol L^−1^ at 9 cm and then declined with depth to reach 1.5 mmol L^−1^ at the bottom of the core. Accordingly, sediment color changed to black and (deeper in the core) grey indicating reducing conditions (Fig. S1a,b). In all three replicates, the majority of methane oxidation occurred in the top 4 cm of the sediment with rates up to 196 nmol cm^−3^ d^−1^ in the top (0–1 cm) sediment layer (Fig. 2c). This activity showed no match with sulfate reduction (Fig. 2d), neither in the profile, nor in magnitude, and suggests that it was coupled to MOx. Methane oxidation reached a minimum (~ 0.4 nmol cm^−3^ d^−3^) at 5–6 cm, below which rates increased again (see insert in Fig. 2c) reaching a maximum of 4.8 nmol cm^−3^ d^−1^ at 7–8 cm (Fig. 2c). The double peaking of methane oxidation suggests a change from an aerobic to an anaerobic methane oxidation pathway likely coupled to sulfate reduction below 6 cm, i.e., below the bio-irrigation activity of the tubeworms. Methane oxidation declined below the second peak to values ~ 1 nmol cm^−3^ d^−3^ at the bottom of the core. Sulfate reduction was low (< 3 nmol cm^−3^ d^−3^) in the top 0–1 cm, but steeply increased in all three replicates reaching values between 11 and 23 nmol cm^−3^ d^−3^ at 2–3 cm (Fig. 2d). Below 3 cm, sulfate reduction steadily declined reaching values ~ 1 nmol cm^−3^ d^−3^ at 10 cm, which remained consistently low down to the bottom of the core. The decoupling of methane oxidation and sulfate reduction in the surface sediment suggest that sulfate reduction was coupled to organic matter degradation in the top 6 cm, while part of it was likely also coupled to anaerobic methane oxidation (AOM) below 6 cm.

The sediment from the bacterial mat field (MUC 12) showed steep geochemical gradients in the top 3–4 cm of the sediment: pore water sulfate and sulfide concentration declined from 28 to 2 and from 6.5 to 0.8 mmol L^−1^, respectively, while total alkalinity increased from 2.5 to 35 mmol L^−1^ (Fig. 2e,f). Methane peaked with concentrations ~ 11 mmol L^−1^ at 2–4 and 28.5 cm and varied between 2 and 5 mmol L^−1^ in other depths with no clear trend (Fig. 2f). It is likely that measured concentrations were below in-situ levels and that the true methane profile was blurred due to degassing after sample recovery from depth. Degassing was clearly noticeable during core handling (Fig. S1c,d). Methane oxidation was low at the surface (< 13 nmol cm^−3^ d^−1^) and steeply increased in all three replicates to a maximum of up to 181 nmol cm^−3^ d^−1^ between 2 and 5 cm (Fig. 2g). Below the peaks, methane oxidation in all three replicates sharply declined and reached values around 1–4 nmol cm^−3^ d^−1^ below 7 cm. Profiles of all three sulfate reduction samples showed a general alignment with methane oxidation (Fig. 2h), suggesting a coupling to AOM. However, sulfate reduction was about two times higher than methane oxidation in the surface sediment (maximum 408 nmol cm^−3^ d^−1^) and therefore likely also coupled to other processes, most reasonably organic matter degradation.

### Foraminiferal isotopic signatures

**Figure 3:**
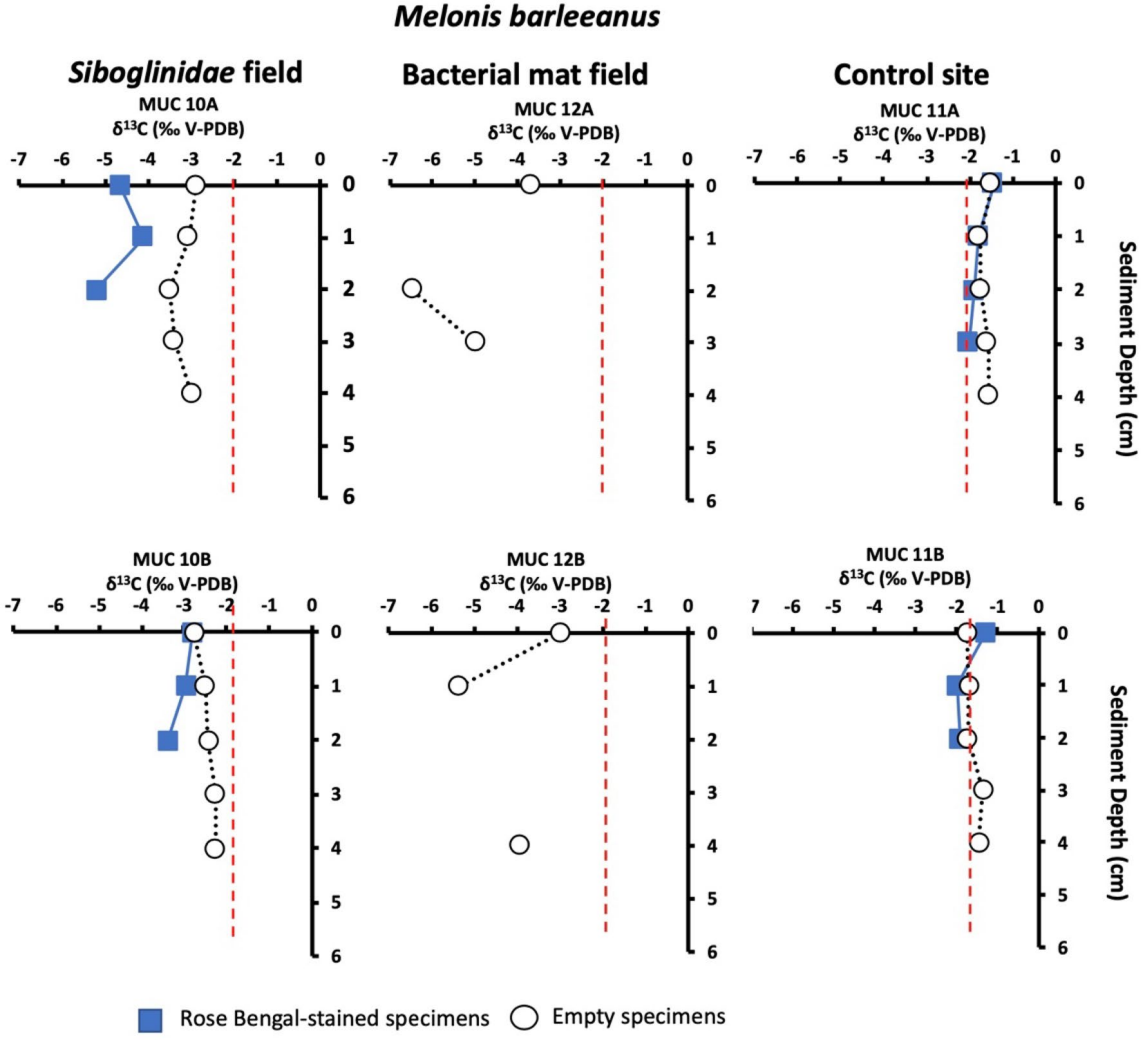
Carbon isotope values (δ^13^C) of *Melonis barleeanus* in sediment from the *Siboglinidae* field (MUC 10A and MUC 10B), bacterial mat field (MUC 12A and MUC 12B), and control site (MUC 11A and MUC 11B). The vertical red line indicates the δ^13^C minimum value for non-seep conditions^34^.

**Figure 4:**
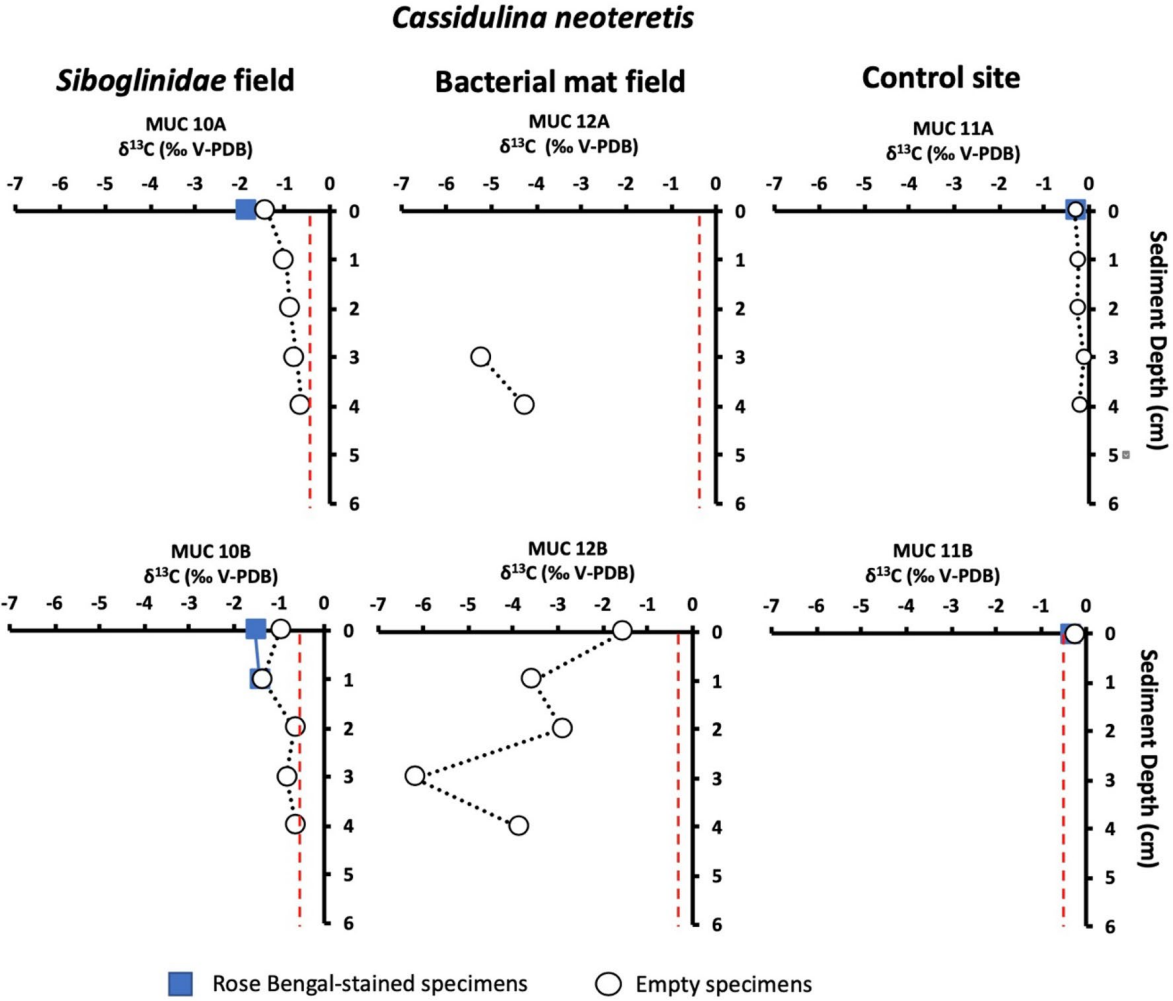
Carbon isotope values (δ^13^C) of *Cassidulina neoteretis* in sediment from the *Siboglinidae* field (MUC 10A and MUC 10B), bacterial mat field (MUC 12A and MUC 12B), and control site (MUC 11A and MUC 11B). The vertical red line indicates the δ^13^C minimum value for conditions^34^.

**Figure 5:**
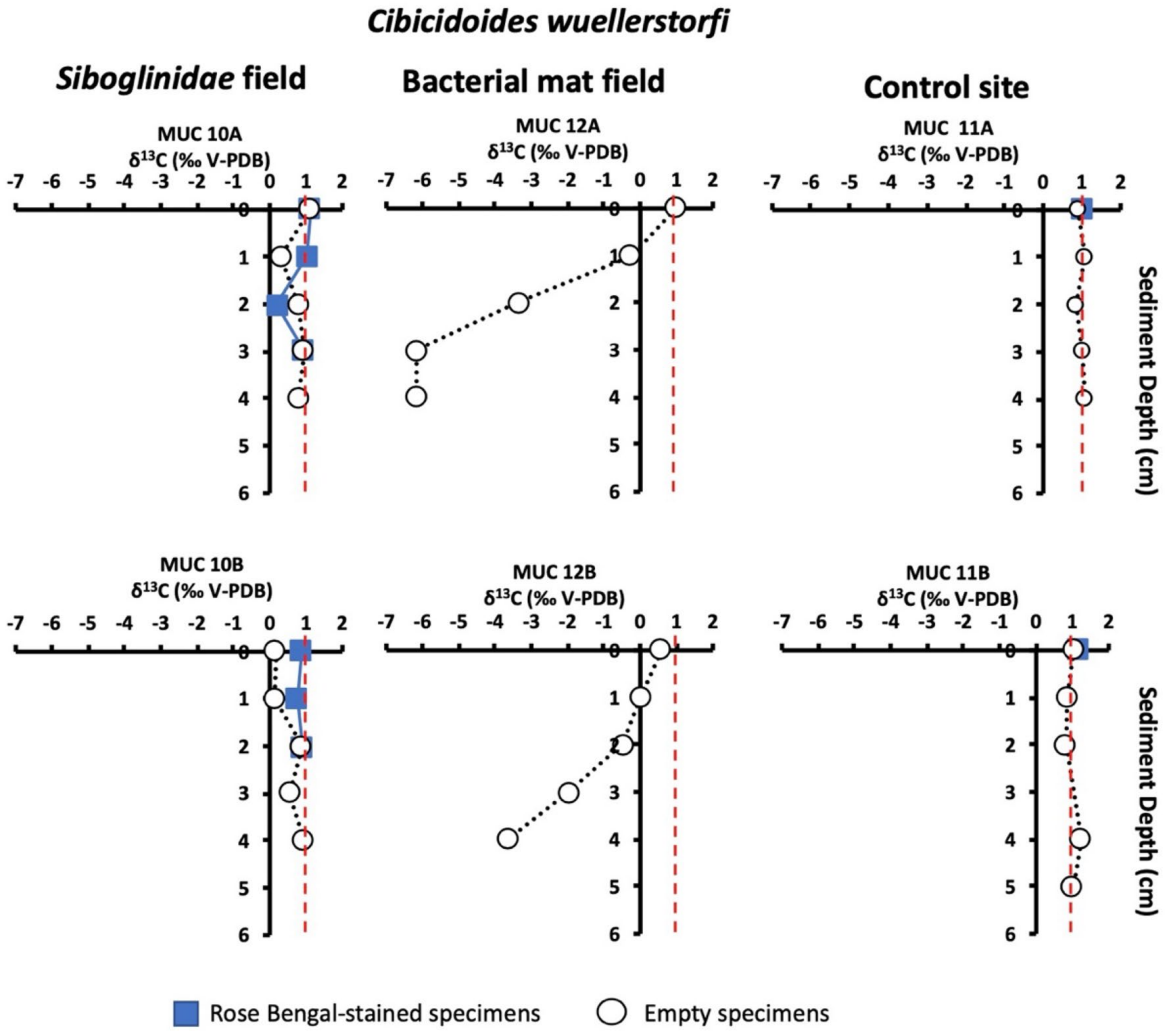
Carbon isotope values (δ^13^C) of *Cibicidoides wuellerstorfi* from the *Siboglinidae* field (MUC 10 and MUC 10B), bacterial mat field (MUC 12A and MUC 12B), and control site (MUC 11A and MUC 11B). The vertical red line indicates the δ^13^C minimum value for non-seep conditions^34^.

**Figure 6:**
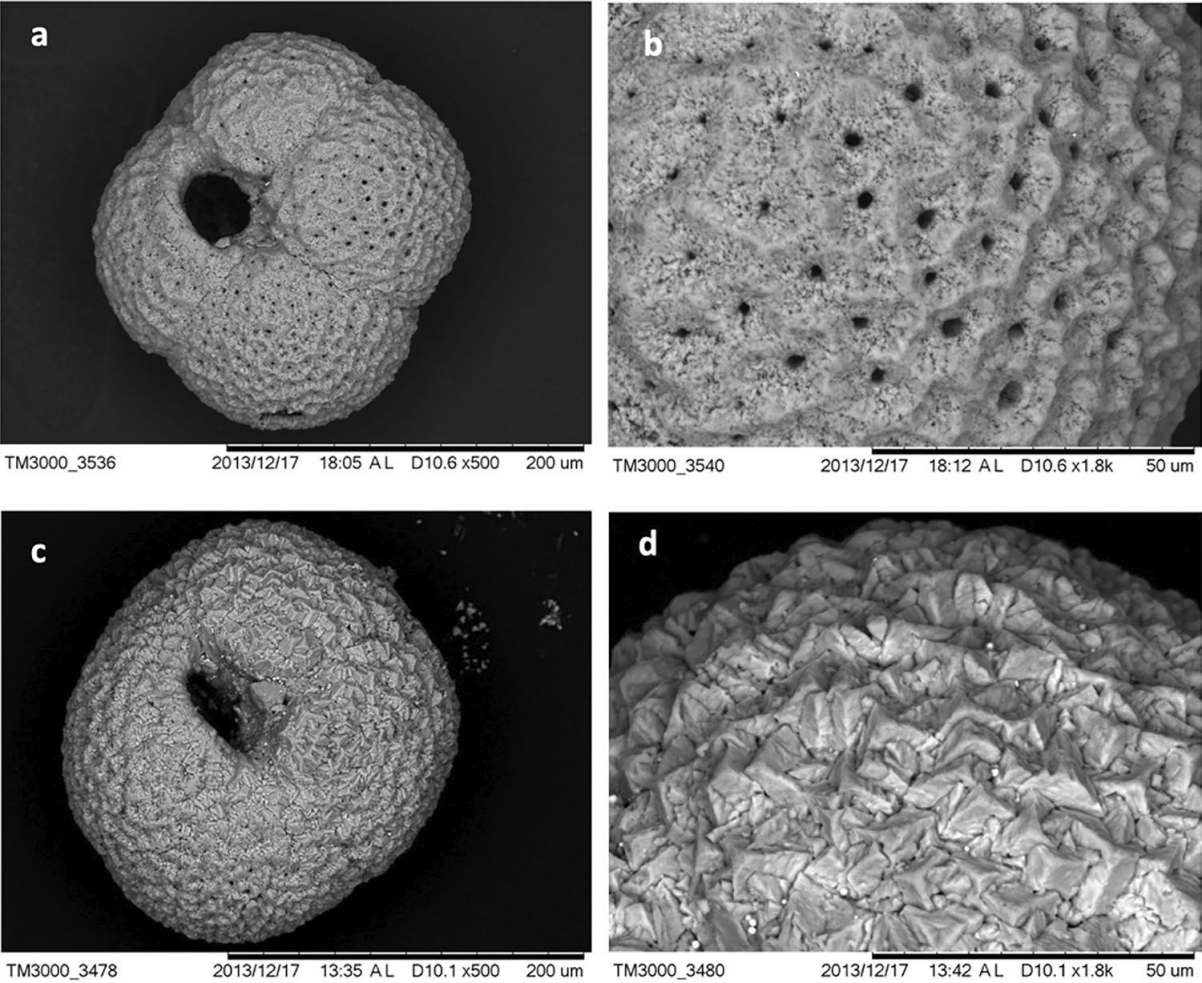
Scanning electron microscopy (SEM) micrographs of *N. pachyderma* from *Siboglinidae* field MUC 10B 4–5 cm depth (**a**,**b**) and bacterial mat field MUC 12B 3–4 cm depth (**c**,**d**). Micrographs (**c**) and (**d**) show authigenic overgrowth on the outer surface of the test, while (**a**,**b**), show a pristine shell with no coating.

Carbon isotopic signatures (δ^13^C) of Rose Bengal stained (RB-stained) foraminiferal specimens from the *Siboglinidae* field (MUC 10) showed the lowest values (Table S2) for the benthic foraminiferal species *M. barleeanus* and *C. neoteretis,* reaching values as low as − 5.2 and − 1.8‰, respectively, as compared to non-stained specimens (i.e., empty tests) (Fig. 4). No RB-stained specimens occurred in sediments from the bacterial mat field (MUC 12). At this station, the lowest δ^13^C were detected, with values as low as − 6.5, − 6.2, and − 6.2‰ in empty specimens of *M. barleeanus*, *C. wuellerstorfi,* and *C. neoteretis*, respectively (Figs. 3, 4, 5, 6a; Table S2). Similarly, empty specimens of the planktonic foraminiferal species *N. pachyderma* show the lowest values, reaching − 6.2‰ (Table S2).

It is notable that the δ^13^C of RB-stained specimens of *M. barleeanus*, C. *wuellerstorfi,* and *C. neoteretis* were lower in samples from the *Siboglinidae* field MUC 10A compared to MUC 10B (Figs. 3, 4, 5). The carbon isotopic signature of RB-stained *M. barleeanus* and *C. wuellerstorfi* displayed a wide range of values (0.2–1.1‰, and − 2.8 to − 5.2‰, respectively; Figs. 3, 5). Overall, RB-stained *M. barleeanus* had lower δ^13^C values in comparison to empty specimens of its conspecifics from the same interval (Fig. 3). Both RB-stained and empty tests of *M. barleeanus* were more depleted in ^13^C in deeper parts of the sediment. The most pronounced negative excursion in δ^13^C was recorded in empty specimens *M*. *barleeanus* from the bacterial mat field (MUC 12A; Fig. 3) reaching − 6.5‰. SEM investigations of empty specimens of *N. pachyderma* (MUC 12B) revealed authigenic overgrowth of carbonate on the surface of their tests (Fig. 6c,d).

**Figure 7:**
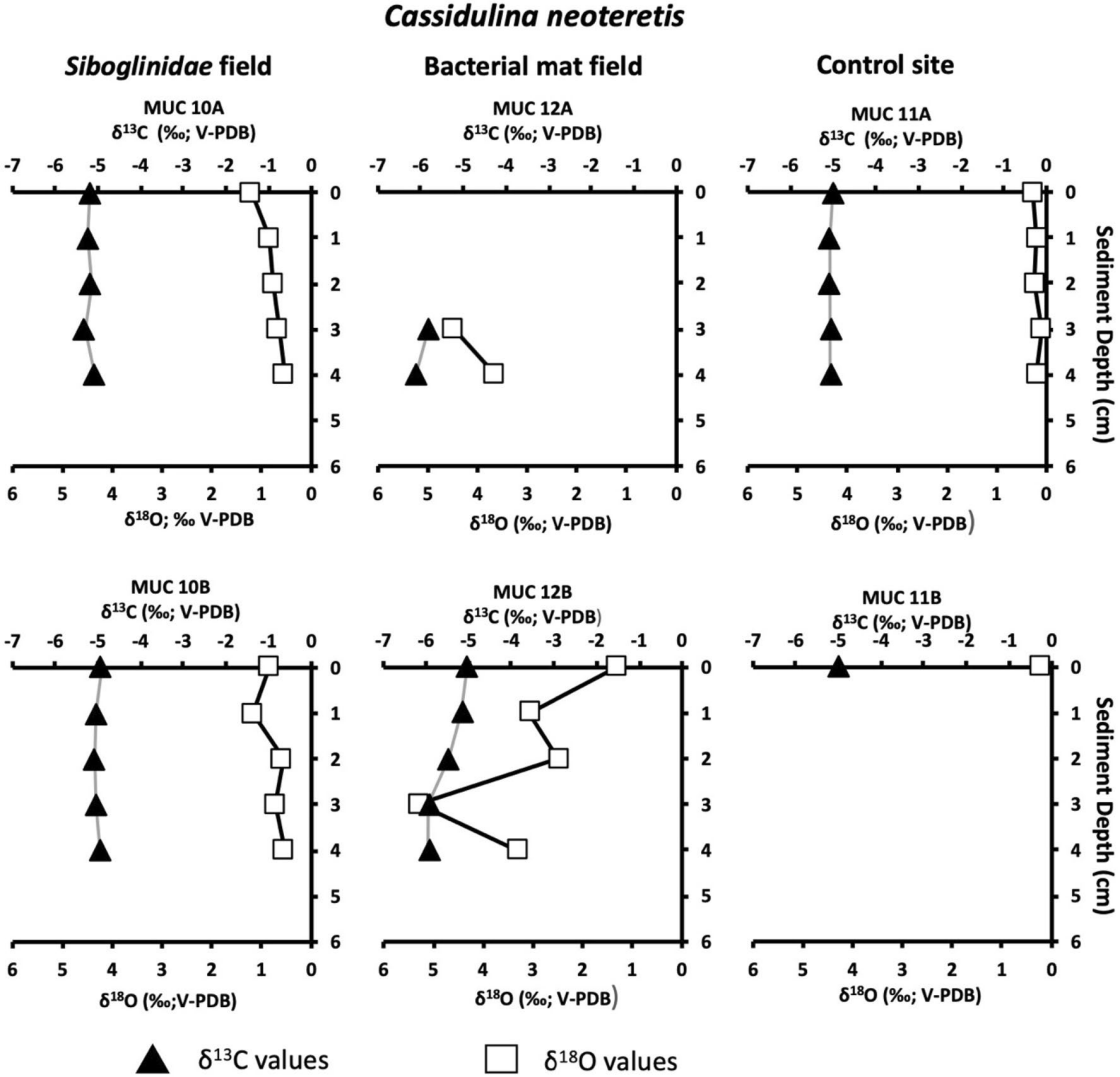
Oxygen isotope values (δ^18^O) of empty tests of *Cassidulina neoteretis* in sediment from the *Siboglinidae* field (MUC 10A and MUC 10B), bacterial mat field (MUC 12A and MUC 12B), and control site (MUC 11A and MUC 11B).

Highest oxygen isotopic values (δ^18^O) were recorded in empty specimens of *C. neoteretis* from the bacterial mat field MUC 12A, reaching 5.2‰ (Table S2; Fig. 7). The δ^18^O values recorded in the RB-stained foraminiferal assemblages from the *Siboglinidae* field MUC 10 (corrected for vital effects; see “Methods”) varied between 4.2–4.4‰ for *M. barleeanus* (Table S2), 4.2–4.4‰ for *C. wuellerstorfi*, and 4.2–4.3‰ for *C. neoteretis*. At the control site, values for RB-stained foraminifera ranged between 4.2–4.4, 4.2–4.4‰, and 4.1–4.2‰. The δ^18^O values for empty foraminifera from the *Siboglinidae* field ranged between 4.3–4.6‰, 4.4–4.6‰, and 2.2–4.6‰ for *M. barleeanus*, *C. wuellerstorfi,* and *C. neoteretis,* respectively. At the bacterial mat site MUC 12, the δ^18^O signatures were between 4.1–4.4‰, 4.2–4.6‰, and 4.3–5.2‰, respectively. Similarly, the δ^18^O values for the empty conspecifics from the control site ranged 4.2–4.5‰, 4.3–4.5‰, and 4.3–4.4‰ for *M. barleeanus*, *C. wuellerstorfi,* and *C. neoteretis* (Table S2, Fig. 7).

## Discussion

### *Siboglinidae* field MUC 10—moderate methane seepage

Biogeochemical data of the pore water from the *Siboglinidae* field (MUC 10) indicate moderate methane seepage activity^19,35,36^. The bio-irrigation activity of the *Siboglinidae* tubeworms might cause oxidation of the top layer of the sediment^37^ and potentially presence of free oxygen, resulting in the consumption of methane by aerobic methanotrophic microorganisms (top 4 cm of the sediment; Fig. 2). As a result of aerobic methane oxidation (MOx), the pore water is likely enriched in methane-derived CO_2_ and HCO_3_^–^^38^ and in microbial biomass, which provides a carbon source for the benthic foraminifera during construction of their tests by biocalcification. To build their tests, benthic foraminifera use carbon from both the ambient DIC pool and intracellular storage (i.e., resulting from respiration and diet^39,40^). Consequently, isotopically light carbon is likely incorporated by the benthic foraminifera, not only as an inorganic carbon from pore water, but also via nutrition (i.e., by consumption of methanotrophic microbes). Presumably, during biocalcification CO_2_ is preferred as CO_2_ diffuses more efficiently across cell membranes compared to HCO_3_^−^ and/or CO_3_^2–^^39^. Irrespective of that, benthic foraminifera have at times been shown to reach low δ^13^C values (down to − 6.9‰ in *Brizalina pacifica*) even in non-seep environments^41^, the fact that δ^13^C values of RB-stained *M. barleeanus* (− 4.1 to − 5.2‰, MUC 10A) from the *Siboglinidae* field are more negative compared to its conspecifics from the control site (− 1.3 to − 2.0‰, MUC 11), as well as compared to other non-seep locations (i.e., normal environments) in the Arctic Ocean (min. − 2‰)^42^ is a clear indication of methane influence. For comparison, previously published δ^13^C values of RB-stained *M. barleeanus* from the ‘Lomvi’ pockmark did not indicate any influence of methane (− 2.0‰)^32^. In this very active ‘Lomvi’ pockmark, studies have shown that methane transport often occurs via mini-fractures, and it is speculated that the gas can escape without affecting the foraminifera^43^. The δ^13^C of RB-stained *C. neoteretis* from the *Siboglinidae* field showed fairly negative values (from − 1.4‰ to − 1.8‰; MUC 10A and -B) compared to the non-seep site MUC 11A, and -B (− 0.3‰) (Fig. 4), and to other non-seep sites from the Arctic Ocean (from − 0.3‰ to − 1‰)^42^. The δ^13^C values of *C. neoteretis* from this study are also considerably lower compared to previously published values (− 0.3‰) from the active ‘Lomvi’ pockmark^32^ (see above).

The δ^13^C of calcareous benthic foraminifera is determined by vital effects i.e., species-specific intracellular metabolic processes^34,44^ and biogeochemical conditions of their microhabitat including organic matter and dissolved inorganic carbon content^45,46^. Vital effects can cause differences in the δ^13^C values of ~ 1–2‰ between specimens from the same species. In the present study, δ^13^C vary from − 5.2 to − 1.3‰ between specimens of RB-stained *M. barleeanus* from the *Siboglinidae* field MUC 10 and from the control site MUC 11. The differences between RB-stained specimens of *C. neoteretis* range from − 0.3 to − 1.8‰. These large differences in δ^13^C values between methane-influenced sites and non-methane sites greatly exceeds values of vital effects and thus cannot be attributed to vital effects alone. We suggest that the major factor controlling the δ^13^C in the foraminiferal tests of RB-stained *M. barleeanus* and *C. neoteretis* in the seep samples from the *Siboglinidae* field MUC 10 comes from microhabitat effects related to presence or absence of methane.

**Figure 8:**
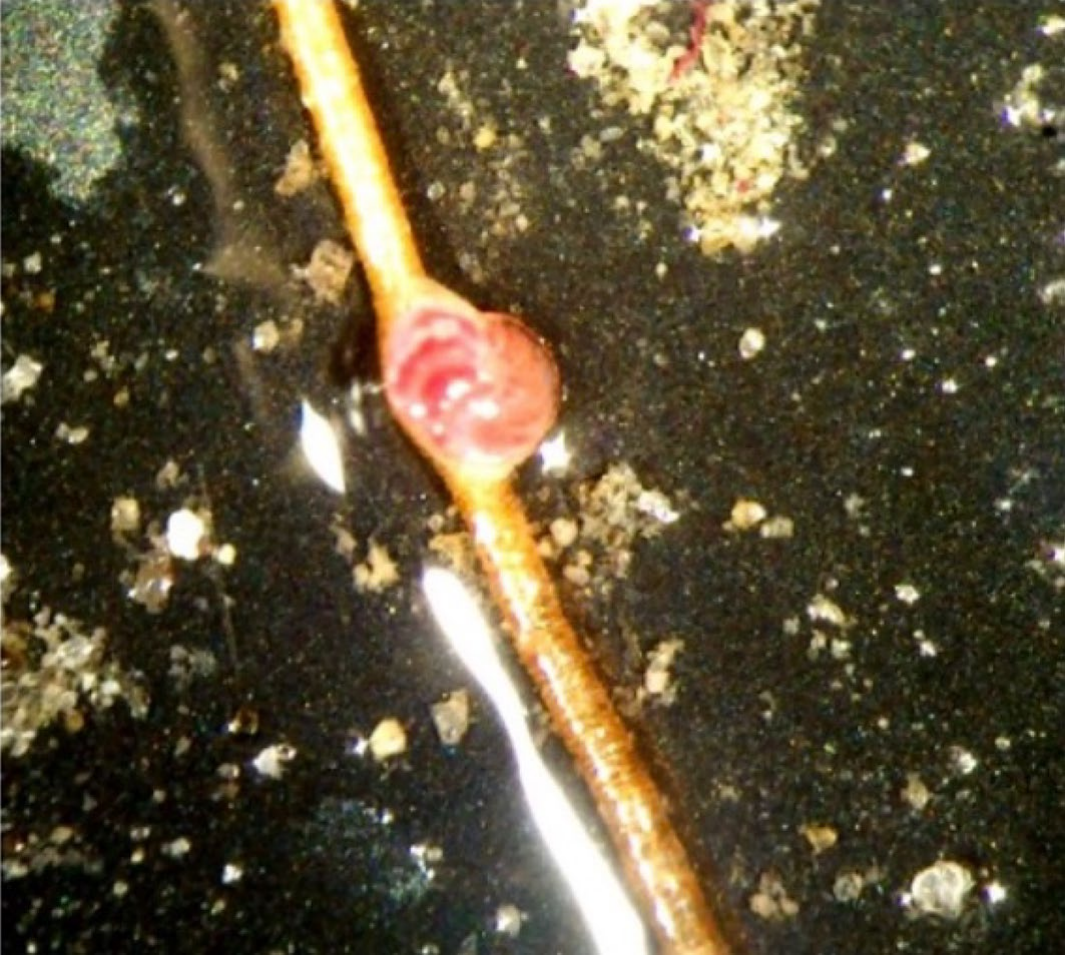
Rose Bengal stained *Cibicidoides wuellerstorfi* attached to a *Siboglinidae* tube; *Siboglinidae* field MUC 10A. Photo: K. Sztybor.

The δ^13^C of RB-stained *C. wuellerstorfi* from the *Siboglinidae* field vary between 0.1 and 1.1‰ (MUC 10A and -B), which is similar to the δ^13^C values of their conspecifics from the control site (from 1.1 to 1.2‰; MUC 11A and -B), and within the range of 'normal' values for the species^21^. Thus, there is no considerable influence of carbon-derived methane on their isotopic signature. This 'normal' carbon isotopic signature is probably related to the epibenthic lifestyle of *C. wuellerstorfi*. The species tends to attach itself to structures extending above the seafloor, e.g., tubes of *Siboglinidae* worms^21,47,48^. They do so to avoid hostile environmental conditions, such as oxygen depletion and toxicity of sulfide, both common at cold seeps^48,49^ (Fig. 2). In the *Siboglinidae* field, samples showed specimens of *C. wuellerstorfi* attached to *Siboglinidae* tubes (Fig. 8). However, due to the absence of hostile environmental conditions, we suggest that *C. wuellerstorfi* is more likely attached to the tubes to support its filter-feeding behavior.

A laboratory culturing experiment performed by Wollenburg et al.^50^ showed that artificially injecting ^13^C-enriched methane to the water altered not only the δ^13^C signatures of the ambient DIC pool, but also the δ^13^C of the foraminiferal offspring of the epifaunal species *C. wuellerstorfi* and the shallow infaunal species *C. neoteretis*. These findings indicate that the δ^13^C values of *C. wuellerstorfi* can become more negative with low δ^13^C_DIC_ in the ambient water. The experiments resulted in mean δ^13^C values of − 1.4‰ for *C. wuellerstorfi* and − 2.2‰ for *C. neoteretis* under controlled culturing conditions^50^. Since the δ^13^C measured in *C. neoteretis* (offspring) has values similar to those obtained from an in-situ reference site from the Håkon Mosby Mud Volcano^21^, the authors suggested that the δ^13^C of this shallow infaunal species mainly reflect their dietary preferences, i.e., feeding on bacteria^50^, whereas the epifaunal species *C. wuellerstorfi* reflects the δ^13^C of the bottom water DIC^50^. Thus, the δ^13^C signatures of *M. barleeanus*, *C. neoteretis,* and *C. wuellerstorfi* from the same sample might reflect the different microhabitat preferences of these species. Foraminifera that calcify deeper in the sediment (intermediate infaunal and deep-infaunal species), as for example *M. barleeanus,* often have low isotopic values when compared to shallow infaunal or epifaunal species, such as *C. neoteretis* or *C. wuellerstorfi*, respectively^44,45^. Our results from the *Siboglinidae* field (MUC 10) indicate that the proportion of carbon from methane in the ambient bottom water was not sufficient to considerably affect the isotopic signature of the epifaunal *C. wuellerstorfi* in comparison to the species that live deeper in the sediment, i.e., *M. barleeanus* and *C. neoteretis* that are more susceptible to the effects of methane. These infaunal species are probably affected by feeding on the ^13^C-depleted methanotrophic microbial communities within the sediment and from incorporating ^13^C-depleted DIC from the pore water during calcification. Food sources of ^13^C-depleted microbes (archaea, bacteria) can contribute to up to a 5 to 6‰ decrease of the δ^13^C values of foraminiferal tests at seep sites^22^.

### Bacterial mat field MUC 12—strong methane seepage

It has been suggested that benthic foraminifera do not calcify in environments influenced by strong methane seepage with hostile concentrations of H_2_S^51^ and consequently their δ^13^C values do not record methane seepage. Our results on the pore water biogeochemistry of the bacterial mat field MUC 12 indicate a strong methane seepage regime, which is dominated by AOM and sulfate reduction and features high concentrations of H_2_S just below the sediment surface (Fig. 2). Although foraminifera have a high tolerance to short-term exposure to H_2_S (up to 21 days), the prolonged exposure to H_2_S (> 66 days, with final concentration of H_2_S 12 μM), results in a significant reduction of the living population^52^. Indeed, no RB-stained foraminifera are found in samples from the bacterial mat field MUC 12A and -B. Nevertheless, the δ^13^C of empty tests from the bacterial mat reached values as low as − 6.5‰ (*M. barleeanus*) and − 6.2‰ (*C. neoteretis*) (Figs. 3, 4, 7; Table S2), and even − 6.2‰ for *C. wuellerstorfi* (Fig. 5). These values are considerably lower compared to δ^13^C values of empty tests from its conspecifics from the control site MUC 11A and -B, which show ‘normal’ values, and are also much lower than in empty foraminiferal shells from the *Siboglinidae* field MUC 10A and -B (Figs. 3, 4, 5). We assume the low δ^13^C values are related to the formation of MDAC related to AOM, which may severely overprint the initial isotopic signatures of the foraminiferal tests and further indicates that the process is of minor importance or even absent at the *Siboglinidae* field MUC 10B. In support of this hypothesis, the SEM investigation of the planktonic foraminiferal species *N. pachyderma* from the bacterial mats field MUC 12B revealed signs of authigenic precipitation of carbonate on the outer surface (Fig. 7c,d), and the δ^13^C signature is considerably more negative (− 0.9 to − 4.2‰) (Table S2) compared to ‘normal’ values of the species in surface water environments (− 0.5‰)^53^.

AOM is a strong contributor to authigenic carbonate overgrowth due to its production of HCO_3_^−^ and increase in alkalinity^12,18^ (and references therein). Overprinting by authigenic carbonate on foraminiferal shells can cause a lowering of the δ^13^C values of > 10‰^12,18^. In the bacterial mat field MUC 12 samples, overgrowth was detected at relatively shallow sediment depth, i.e., 2–3 cm below the sediment surface (MUC 12A). Hence, the measured low values are most likely the result of a minor degree of a very recent overgrowth and are therefore less depleted in ^13^C compared to values previously recorded in Vestnesa Ridge studies^12,18^.

The δ^18^O ratio in calcareous foraminiferal tests is influenced by several factors, including bottom water temperature, isotopic composition of the ambient seawater, and vital effects^54^, but can also be changed by diagenetic coating of foraminiferal tests from MDAC, which can increase the δ^18^O values in both benthic and planktic foraminifera^8,55,56^. Thus, foraminiferal tests of low δ^13^C values from methane-derived carbon might also record higher δ^18^O values^12^ (and references therein). Our data show that the δ^18^O signature in empty tests of *C. neoteretis* from the bacterial mat field (MUC 12A and -B) display low δ^13^C values and a high δ^18^O signature (up to 5.2‰). There was no such pattern in *C. neoteretis* from the *Siboglinidae* field MUC 10 or the control site MUC 11 (Fig. 6). We suggest that the higher ^18^O results from the precipitation of ^18^O-enriched MDAC in line with results from Cook et al.^56^. Similarly, δ^18^O in *N. pachyderma* show relatively high values (3.1–4.1‰)^53^ (Table S2). In accordance with these observations, authigenic carbonates from seep sites in the ‘Lomvi’ pockmark also displayed relatively high δ^18^O values (4.5 to 5.9‰)^12^. Given the more positive values, *C. neoteretis* might have a high predisposition for authigenic overgrowth, likely due to its test structure^18^, which may explain why only this species showed higher δ^18^O values compared to other species from the same samples.

## Conclusion

The δ^13^C values measured in both RB-stained benthic foraminifera and empty tests of both planktonic and benthic foraminifera from Vestnesa Ridge together with biogeochemical datasets of pore water conditions showed a large degree of variation between different habitats (*Siboglinidae* field, bacterial mat field, and control site). At the *Siboglinidae* field MUC 10 with moderate seepage of methane, dominance of aerobic methane oxidation (MOx), and low concentrations of sulfide, live benthic foraminifera (RB-stained) incorporate methane-derived carbon. We propose that methane derived carbon was incorporated via feeding on methane-oxidizing bacteria and/or by direct intake of CO_2_ in dissolved inorganic carbon produced from MOx. The effect, however, differed between species: the epifaunal species *Cibicidoides wuellerstorfi* appeared to be less susceptible to methane influence, while the intermediate infaunal species *Melonis barleeanus* responded more strongly by reaching δ^13^C values down to − 5.2‰. In sediments from the bacterial mat field MUC 12 with strong methane seepage, high activity of anaerobic oxidation of methane and sulfate reduction produced high levels of sulfide and total alkalinity, which killed living specimens and lead to the lowest δ^13^C values recorded in dead specimens due to postmortem MDAC overgrowth, respectively. Overgrowth may have started the coating of the fossil foraminiferal tests at relatively shallow depth in the sediment (2–3 cm), causing δ^13^C signature shifts of tests towards low values (down to − 6.5‰ for fossil *M. barleeanus).* Higher δ^18^O values in fossil *C. neoteretis* (5.1‰) from the bacterial mat field MUC 12 combined with low δ^13^C values (− 6.2‰) also indicate MDAC coatings of their tests.

Fossil records derived from benthic foraminifera thus reflect the cumulative history of methane seepage covering the lifespan of the organisms, during which methane-derived carbon may be incorporated, as well as post-mortem processes, such as shell overgrowth by MDAC. Therefore, in the context of palaeoceanographic studies, the use of δ^13^C signatures from foraminiferal shells as a paleo-methane indicator requires the consideration of MDAC coatings to separate between processes occurring during and after the lifetime of a benthic foraminifera.

## Methods

### Sediment sampling

Sediment samples were collected from a pockmark on Vestnesa Ridge, NW Svalbard margin in August 2011 during the POS419 expedition of the RV Poseidon. Using a TV-guided multicorer, sediment samples were taken from a *Siboglinidae* field (i.e. sediments covered by chemosymbiotic tubeworms), from a sulfur-bacterial mat field, and from far outside of the pockmark as a control site, where no methane seepage occurs (Table S1). The TV-guided multicorer system enables visual localization of active methane seeps based on the presence of cold-seep related structures, such as bacterial mats and authigenic carbonate crusts for targeted, designated sampling spots. The multicorer collected 6 cores of 10 cm in diameter at each location. After recovery of the multicorer, two cores (labelled A, B) were selected from each site for the study of foraminifera and subsampled onboard into 1-cm thick horizontal slices down to 10 cm core depth. The samples were transferred into plastic containers, and stained with Rose Bengal-ethanol solution following the FOBIMO protocol (2 g/L)^57^. Samples were kept onboard in a dark cool room at + 4 °C until further processing. A third core was sectioned in 1, 2, 3, and 5 cm increments (from top to bottom) for sediment pore water analyses. A fourth core was sectioned in 1, 2, 3, and 5 cm increments (from top to bottom) for sediment methane analyses. A fifth core was subsampled with a total of six mini polycarbonate cores (inner diameter 26 mm, length 30 cm) for the determination of methane concentration, methane oxidation, and sulfate reduction. All sediment sampling procedures were conducted at + 4 °C inside an environmental room.

### Pore water analyses

Pore water was extracted onboard at + 4 °C using a low‐pressure squeezer (argon at 1–5 bar). While squeezing, pore water was filtered through 0.2 μm cellulose acetate nuclepore filters and collected in argon‐flushed recipient vessels. Pore water samples were subjected to geochemical analyses for total sulfides, total alkalinity, sulfate- and methane concentrations.

### Sulfide, total alkalinity and sulfate measurements

Onboard, the collected pore water samples were analyzed for their content of dissolved total sulfides (in the following referred to as “sulfide”)^58^. A 1 mL sample was added to 50 μL of zinc acetate solution. Subsequently, 10 μL of N,N‐dimethyl‐1,4‐phenylenediamine‐dihydrochloride color reagent solution and 10 μL of the FeCl_3_ catalyst were added and mixed. After 1 h of reaction time, the absorbance was measured at 670 nm. Total alkalinity (TA) was determined by direct titration of 1 mL pore water with 0.02 M HCl using a mixture of methyl red and methylene blue as an indicator and bubbling the titration vessel with argon gas to strip CO_2_ and hydrogen sulfide. The analysis was calibrated using IAPSO seawater standard, with a precision and detection limit of 0.05 mmol L^−1^. Pore water samples for sulfate (SO_4_^2−^) analyses were stored in 2‐mL glass vials at + 4 °C and analyzed onshore. Sulfate was determined by ion chromatography (Metrohm, IC Compact 761). Analytical precision based on repeated analysis of IAPSO standards (dilution series) was < 1%.

### Methane measurements

According to Sommer et al.^59^ methane concentrations in sediment cores were determined in 1-cm intervals down to a depth of 6 cm followed by 2-cm intervals down to 12 cm, 3-cm intervals down to 18 cm and 5-cm intervals deeper than 18 cm. From each depth horizon, a 2-mL sub-sample was transferred into a septum-stoppered glass vial (21.8 mL) containing 6 mL of saturated NaCl solution and 1.5 g of NaCl in excess. The volume of headspace was 13.76 mL. Within 24 h, the methane concentration in the headspace was determined using a Shimadzu GC 14A gas chromatograph fitted with a flame ionization detector and a 4-m × 1⁄8-in. Poraplot Q (mesh 50/80) packed column. Prior to the measurements the samples were equilibrated for 2 h on a shaking table. Precision to reproduce a methane standard of 9.98 ppm was 2%.

### Microbial methane oxidation rates

On board, radioactive methane (^14^CH_4_ dissolved in water, injection volume 15 µL, activity ~ 5 kBq, specific activity 2.28 GBq mmol^−1^) was injected into three replicate mini cores at 1-cm intervals according to the whole-core injection method^60^. The mini cores were incubated at in-situ temperature for ~ 24 h in the dark. To stop bacterial activity, the sediment cores were sectioned into 1-cm intervals and transferred into 50-mL crimp glass vials filled with 25 mL sodium hydroxide (2.5% w/w). After crimp-sealing, glass vials were shaken thoroughly to equilibrate the pore-water methane between the aqueous and gaseous phase. Control samples were first terminated before addition of tracer. In the home laboratory, methane oxidation rates and methane concentrations in the sample vials were determined according to Treude et al.^61^.

### Microbial sulfate reduction rates

Sampling, injection, and incubation procedures were identical to methane oxidation samples. The injected radiotracer was carrier-free ^35^SO_4_^2−^ (dissolved in water, injection volume 6 µL, activity 200 kBq, specific activity 37 TBq mmol^−1^). To stop bacterial activity after incubation, sediment cores were sectioned into 1-cm intervals and transferred into 50 mL plastic centrifuge vials filled with 20 mL zinc acetate (20% w/w) and frozen. Control sediment was first terminated before addition of tracer. In the home laboratory, sulfate reduction rates were determined according to the cold-chromium distillation method^62^.

### Foraminiferal analyses

Rose-Bengal stained samples were sieved over a 100-µm sieve. The > 100-µm fraction was kept wet and further examined under reflected-light microscopy. All benthic foraminiferal individuals that stained dark magenta and were fully filled with cytoplasm were considered to be ‘living’ foraminifera i.e., live + recently dead individuals, still containing cytoplasm. Foraminifera showing no colorization were considered as unstained, empty (dead) individuals. The foraminifera were wet picked, sorted by species and placed on micropaleontology slides.

### Isotope analyses

For carbon (δ^13^C) and oxygen (δ^18^O) stable isotope analyses, both Rose Bengal stained and unstained (empty) specimens of benthic foraminiferal species *Melonis barleeanus*, *Cassidulina neoteretis* and *Cibicidoides wuellerstorfi,* and empty specimens of the planktic foraminiferal species *Neogloboquadrina pachyderma* were picked. When present, approximately 10 specimens of each species were taken from each sample. Only pristine and transparent, and clean tests were picked. Empty tests were obtained from the same samples as the Rose-Bengal stained foraminifera. No replicate measurements for isotope ratios were made due to low amounts of foraminiferal material available. Samples were cleaned using pure ethyl alcohol in an ultrasonic bath, following the protocol from Sztybor and Rasmussen^12^ Isotopic measurements were performed at the Isotope Geochemistry Facility at Woods Hole Oceanographic Institution (WHOI). Data are reported in standard notation (δ^13^C, δ^18^O), according to the Pee Dee Belemnite (PDB) standard. Reported precision was estimated to be ± 0.07‰ for δ^13^C and ± 0.15‰ for δ^18^O by measuring the certified standard NBS-19. The δ^18^O values were corrected for vital effects as follows: + 0.4‰ for *M. barleeanus*^14^ and + 0.64‰ for *C. wuellerstorfi*^63^*. Neogloboquadrina pachyderma*, two specimens from intervals 0–1 cm and 4–5 cm from each core were selected and investigated using Scanning Electron Microscopy (SEM). To make our data comparable with other studies on live foraminifera, we deliberately used the most widely used staining method, the Rose Bengal. Since Rose Bengal indicates both live and dead cytoplasm, even weeks to months after the death of an individual^32,64^, we nevertheless refer here to Rose Bengal stained foraminifera as ‘live’ specimens, and empty, unstained tests as dead specimens.

## Supplementary Information

Supplementary Information.
