# Supplementary material for: Influence of methane seepage on isotopic signatures in living deep-sea benthic foraminifera, 79° N
Source: Sci Rep. 2022 Jan 21;12:1169. doi: 10.1038/s41598-022-05175-1 (PMC8782907; doi:10.1038/s41598-022-05175-1)
Supplement: Supplementary file 1 — Supplementary Information. [file 41598_2022_5175_MOESM1_ESM.docx]

**Influence of methane seepage on isotopic signatures in living deep-sea benthic foraminifera, 79 °N**

Katarzyna Melaniuk ^1*^, Kamila Sztybor ^2^, Tina Treude ^3, 4^, Stefan Sommer ^5^, Tine L. Rasmussen^1^

1. Centre of Arctic Gas Hydrate, Environment and Climate CAGE, Department of Geosciences, UiT The Arctic University of Norway, 9010 Tromsø, Norway.
2. Akvaplan-niva AS, Fram Centre, 9296 Tromsø, Norway.
3. Department of Earth, Planetary, and Space Sciences, University of California Los Angeles, Los Angeles, USA.
4. Department of Atmospheric and Oceanic Sciences, University of California Los Angeles, Los Angeles, USA.
5. GEOMAR Helmholtz Centre for Ocean Research Kiel, 24148 Kiel, Germany.

*corresponding author

[Katarzyna.Melaniuk@uit.no](mailto:Katarzyna.Melaniuk@uit.no)

**SUPPLEMENTARY INFORMATION**

**Table S1**. Sample coordinates and environmental information.

| **Station**  **POS419** | **Sample ID** | **Coordinates** | **Depth (cm)** | **Environment** | **Date** | **Analyses** |
| --- | --- | --- | --- | --- | --- | --- |
| 675 | MUC 10 | 79° 00,466´N  06°54,279´E | 1241 | *Siboglinidae* field | 25.08.2011 | stable isotopes, SEM  sulfide, SO_4_^2-^, total alkalinity, methane, AOM, sulfate reduction |
| 676 | MUC 11 | 78° 59,774´N  06° 58,064´E | 1191 | Control site, regular marine sediment | 28.08. 2011 | Stable isotopes, SEM |
| 678 | MUC 12 | 79° 00,417´N  06° 54,131´E | 1235 | Bacterial mats | 29.08. 2011 | stable isotopes, SEM;  sulfide, SO_4_^2-^, total alkalinity, methane, AOM, sulfate reduction |

**Table S2.** Stable isotope values (δ^13^C and δ^18^O; ‰) of Rose Bengal- stained (RB) and empty (fossil) tests of benthic foraminiferal species *Melonis barleeanus*, *Cassidulina neoteretis*, *Cibicidoides wuellerstorfi*, and planktonic foraminiferal species *Neogloboquadrina pachyderma* from Vestnesa Ridge *Siboglinidae* field (MUC 10A, MUC 10B), bacterial mat (MUC 12A, MUC 12B) and control site (MUC 11A and MUC 11B). The * represent the δ^18^O values corrected for vital effects: +0.4‰ for *M. barleeanus* and + 0.64‰ for *C. wuellerstorfi.*

|  |  | Rose Bengal-stained | | | Fossil | | |
| --- | --- | --- | --- | --- | --- | --- | --- |
|  | Depth cm | δ^13^C‰ | δ^18^O‰ | δ^18^O‰* | δ^13^C‰ | δ^18^O‰ | δ^18^O‰* |
| **MUC 10A** |  |  |  |  |  |  |  |
|  | 0–1 | -4.65 | 3.95 | 4.35 | -2.88 | 4.16 | 4.56 |
| *M. barleeanus* | 1–2 | -4.10 | 3.91 | 4.31 | -3.05 | 4.18 | 4.58 |
|  | 2–3 | -5.21 | 3.90 | 4.30 | -3.50 | 4.16 | 4.56 |
|  | 3–4 | - | - | - | -3.38 | 4.19 | 4.59 |
|  | 4–5 | - | - | - | -2.99 | 4.05 | 4.45 |
|  |  |  |  |  |  |  |  |
|  | 0–1 | 1.13 | 3.66 | 4.30 | 1.11 | 3.85 | 4.49 |
| *C. wuellerstorfi* | 1–2 | 1.04 | 3.73 | 4.37 | 0.30 | 3.91 | 4.55 |
|  | 2–3 | 0.18 | 3.72 | 4.36 | 0.79 | 3.88 | 4.52 |
|  | 3–4 | 0.93 | 3.79 | 4.43 | 0.94 | 3.83 | 4.47 |
|  | 4–5 | - | - | - | 0.80 | 3.89 | 4.53 |
|  |  |  |  |  |  |  |  |
|  | 0–1 | -1.83 | 4.26 | - | -1.40 | 4.45 | - |
|  | 1–2 | - | - | - | -0.98 | 4.48 | - |
| *C. neoteretis* | 2–3 | - | - | - | -0.87 | 4.43 | - |
|  | 3–4 | - | - | - | -0.75 | 4.54 | - |
|  | 4–5 | - | - | - | -0.62 | 4.35 | - |
|  |  |  |  |  |  |  |  |
|  | 0–1 | - | - | - | -0.10 | 2.69 | - |
| *N. pachyderma* | 1–2 | - | - | - | 0.20 | 3.16 | - |
|  | 2–3 | - | - | - | -0.29 | 3.42 | - |
|  | 3–4 | - | - | - | 0.06 | 2.93 | - |
|  | 4–5 | - | - | - | - | - | - |
| **MUC 10B** |  |  |  |  |  |  |  |
|  | 0–1 | -2.80 | 3.83 | 4.23 | -2.78 | 3.96 | 4.36 |
| *M. barleeanus* | 1–2 | -2.95 | 3.86 | 4.26 | -2.52 | 3.92 | 4.32 |
|  | 2–3 | -3.28 | 3.88 | 4.28 | -2.43 | 3.96 | 4.36 |
|  | 3–4 | - | - | - | -2.27 | 3.97 | 4.37 |
|  | 4–5 | - | - | - | -2.26 | 3.97 | 4.37 |
|  |  |  |  |  |  |  |  |
|  | 0–1 | 0.88 | 3.72 | 4.36 | 0.15 | 3.76 | 4.40 |
| *C. wuellerstorfi* | 1–2 | 0.77 | 3.63 | 4.27 | 0.13 | 3.88 | 4.52 |
|  | 2–3 | 0.87 | 3.58 | 4.22 | 0.87 | 3.75 | 4.39 |
|  | 3–4 | - | - | - | 0.55 | 3.84 | 4.48 |
|  | 4–5 | - | - | - | 0.95 | 3.80 | 4.44 |
|  |  |  |  |  |  |  |  |
|  | 0–1 | -1.54 | 4.26 | 4.26 | -0.97 | 4.21 | - |
| *C. neoteretis* | 1–2 | -1.44 | 4.17 | 4.17 | -1.37 | 4.32 | - |
|  | 2–3 | - | - | - | -0.66 | 4.35 | - |
|  | 3–4 | - | - | - | -0.84 | 4.31 | - |
|  | 4–5 | - | - | - | -0.62 | 4.24 | - |
|  |  |  |  |  |  |  |  |
| **MUC 11A** |  |  |  |  |  |  |  |
|  | 0–1 | -1.42 | 3.87 | 4.27 | -1.46 | 4.05 | 4.45 |
| *M. barleeanus* | 1–2 | -1.77 | 4.01 | 4.41 | -1.75 | 4.08 | 4.48 |
|  | 2–3 | -1.91 | 4.03 | 4.43 | -1.74 | 3.96 | 4.36 |
|  | 3–4 | -2.01 | 4.01 | 4.41 | -1.57 | 4.04 | 4.44 |
|  | 4–5 | - | - | - | -1.55 | 4.12 | 4.52 |
|  |  |  |  |  |  |  |  |
|  | 0–1 | 1.05 | 3.55 | 4.19 | 0.92 | 3.85 | 4.49 |
| *C. wuellerstorfi* | 1–2 | - | - | - | 1.08 | 3.73 | 4.37 |
|  | 2–3 | - | - | - | 0.85 | 3.81 | 4.45 |
|  | 3–4 | - | - | - | 1.02 | 3.69 | 4.33 |
|  | 4–5 | - | - | - | 1.08 | 3.84 | 4.48 |
|  |  |  |  |  |  |  |  |
|  | 0–1 | -0.32 | 4.14 | - | -0.32 | 4.29 | - |
| *C. neoteretis* | 1–2 | - | - | - | -0.23 | 4.35 | - |
|  | 2–3 | - | - | - | -0.27 | 4.36 | - |
|  | 3–4 | - | - | - | -0.11 | 4.33 | - |
|  | 4–5 | - | - | - | -0.22 | 4.34 | - |
|  |  |  |  |  |  |  |  |
| **MUC 11B** |  |  |  |  |  |  |  |
|  | 0–1 | -1.30 | 3.87 | 4.27 | -1.75 | 3.93 | 4.33 |
| *M. barleeanus* | 1–2 | -1.99 | 3.89 | 4.29 | -1.71 | 4.02 | 4.42 |
|  | 2–3 | -1.93 | 3.82 | 4.22 | -1.73 | 3.81 | 4.21 |
|  | 3–4 | - | - | - | -1.37 | 4.11 | 4.51 |
|  | 4–5 | - | - | - | -1.46 | 4.12 | 4.52 |
|  |  |  |  |  |  |  |  |
|  | 0–1 | 1.20 | 3.77 | 4.41 | 1.05 | 3.61 | 4.25 |
| *C. wuellerstorfi* | 1–2 | - | - | - | 0.86 | 3.73 | 4.37 |
|  | 2–3 | - | - | - | 0.83 | 3.59 | 4.23 |
|  | 3–4 | - | - | - | 1.25 | 3.76 | 4.40 |
|  | 4–5 | - | - | - | 1.02 | 3.73 | 4.37 |
|  |  |  |  |  |  |  |  |
| *C. neoteretis* | 0–1 | -0.34 | 4.23 | - | -0.22 | 4.29 | - |
|  |  |  |  |  |  |  |  |
| **MUC 12A** |  |  |  |  |  |  |  |
|  | 0–1 | - | - | - | -3.68 | 3.92 | 4.32 |
| *M. barleeanus* | 1–2 | - | - | - | - | - | - |
|  | 2–3 | - | - | - | -6.48 | 3.70 | 4.10 |
|  | 3–4 | - | - | - | -4.99 | 4.01 | 4.41 |
|  | 4–5 | - | - | - | - | - | - |
|  |  |  |  |  |  |  |  |
|  | 0–1 | - | - | - | 0.98 | 3.99 | 4.63 |
| *C. wuellerstorfi* | 1–2 | - | - | - | -0.27 | 3.67 | 4.31 |
|  | 2–3 | - | - | - | -3.35 | 3.54 | 4.18 |
|  | 3–4 | - | - | - | -6.17 | 3.51 | 4.15 |
|  | 4–5 | - | - | - | -6.17 | 3.67 | 4.31 |
|  |  |  |  |  |  |  |  |
|  | 0–1 | - | - | - | - | - | - |
| *C. neoteretis* | 1–2 | - | - | - | - | - | - |
|  | 2–3 | - | - | - | - | - | - |
|  | 3–4 | - | - | - | -5.22 | 4.99 | - |
|  | 4–5 | - | - | - | -4.25 | 5.24 | - |
|  |  |  |  |  |  |  |  |
|  | 0–1 | - | - | - | -0.92 | 3.26 | - |
| *N. pachyderma* | 1–2 | - | - | - | -3.07 | 3.13 | - |
|  | 2–3 | - | - | - | -3.30 | 3.05 | - |
|  | 3–4 | - | - | - | -4.23 | 4.03 | - |
|  | 4–5 | - | - | - | -3.07 | 3.71 | - |
|  |  |  |  |  |  |  |  |
| **MUC 12B** |  |  |  |  |  |  |  |
|  | 0–1 | - | - | - | -2.98 | 3.79 | 4.19 |
| *M. barleeanus* | 1–2 | - | - | - | -5.32 | 3.69 | 4.09 |
|  | 2–3 | - | - | - | -5.54 | 3.83 | 4.23 |
|  | 3–4 | - | - | - | - | - | - |
|  | 4–5 | - |  | - | -3.90 | 3.84 | 4.24 |
|  |  |  |  |  |  |  |  |
|  | 0–1 | - | - | - | 0.59 | 3.63 | 4.27 |
| *C. wuellerstorfi* | 1–2 | - | - | - | -0.00 | 3.60 | 4.24 |
|  | 2–3 | - | - | - | -0.46 | 3.61 | 4.25 |
|  | 3–4 | - | - | - | -1.97 | 3.61 | 4.25 |
|  | 4–5 | - | - | - | -3.65 | 3.67 | 4.31 |
|  |  |  |  |  |  |  |  |
|  | 0–1 | - | - | - | -1.54 | 4.32 | - |
| *C. neoteretis* | 1–2 | - | - | - | -3.57 | 4.44 | - |
|  | 2–3 | - | - | - | -2.91 | 4.72 | - |
|  | 3–4 | - | - | - | -6.18 | 5.17 | - |
|  | 4–5 | - | - | - | -3.87 | 5.09 | - |


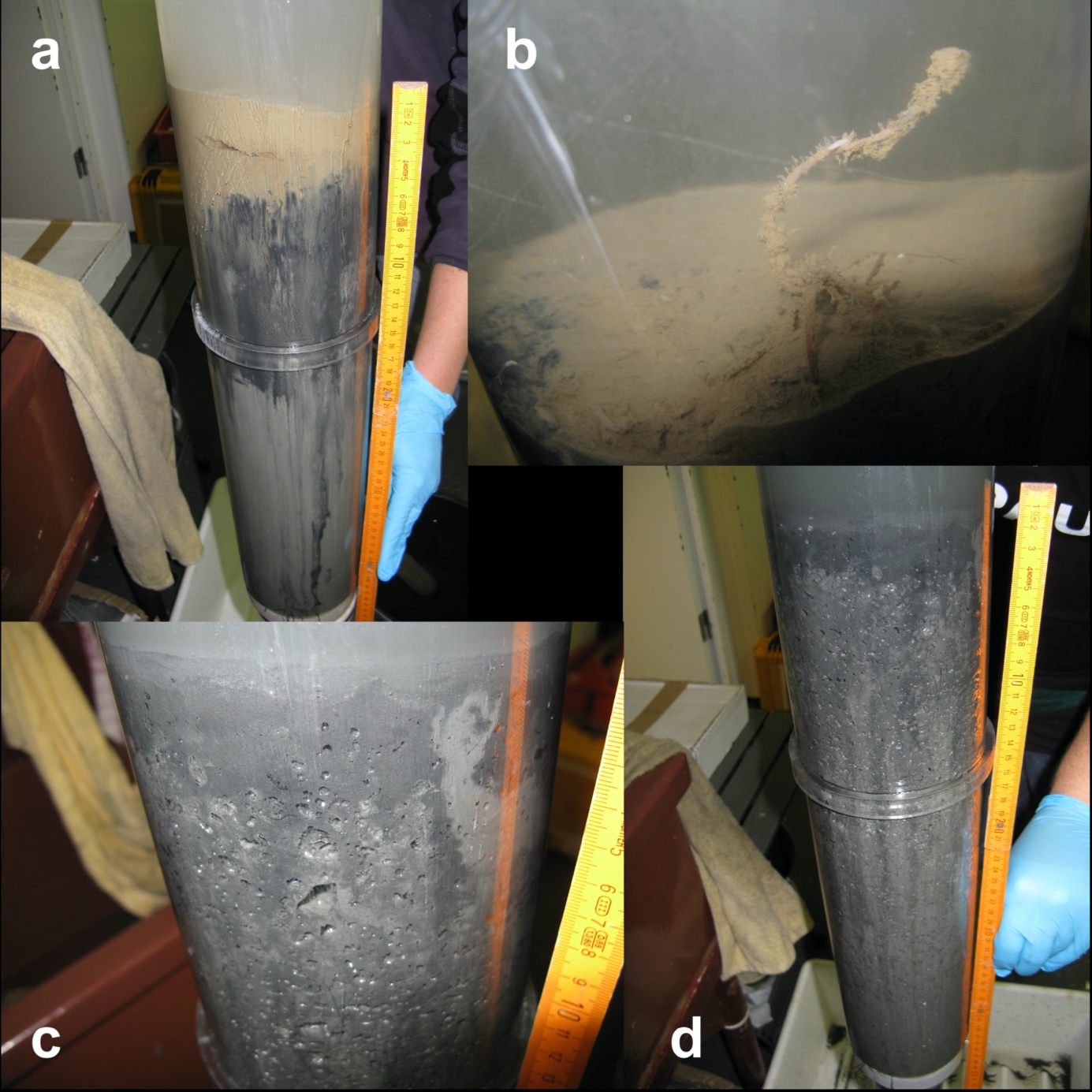


**Figure S1.** Selected sediment cores from MUC 10 (*Siboglinidae* field, a and b) and MUC 12 (bacteria mat, c and d). Note that a *Siboglinidae* tube is shown in b and the degassing of methane is visible in c and d (foamy sediment consistency).
